# Multi-Task Trajectory Prediction Using a Vehicle-Lane Disentangled Conditional Variational Autoencoder

**DOI:** 10.3390/s25144505

**Published:** 2025-07-20

**Authors:** Haoyang Chen, Na Li, Hangguan Shan, Eryun Liu, Zhiyu Xiang

**Affiliations:** The College of Information Science and Electronic Engineering, Zhejiang University, Hangzhou 310027, China; chenhy1997@zju.edu.cn (H.C.); nlee@zju.edu.cn (N.L.); eryunliu@zju.edu.cn (E.L.); xiangzy@zju.edu.cn (Z.X.)

**Keywords:** trajectory prediction, generative model, deep learning, autonomous driving

## Abstract

Trajectory prediction under multimodal information is critical for autonomous driving, necessitating the integration of dynamic vehicle states and static high-definition (HD) maps to model complex agent–scene interactions effectively. However, existing methods often employ static scene encodings and unstructured latent spaces, limiting their ability to capture evolving spatial contexts and produce diverse yet contextually coherent predictions. To tackle these challenges, we propose *MS-SLV*, a novel generative framework that introduces (1) a time-aware scene encoder that aligns HD map features with vehicle motion to capture evolving scene semantics and (2) a structured latent model that explicitly disentangles agent-specific intent and scene-level constraints. Additionally, we introduce an auxiliary lane prediction task to provide targeted supervision for scene understanding and improve latent variable learning. Our approach jointly predicts future trajectories and lane sequences, enabling more interpretable and scene-consistent forecasts. Extensive evaluations on the nuScenes dataset demonstrate the effectiveness of MS-SLV, achieving a 12.37% reduction in average displacement error and a 7.67% reduction in final displacement error over state-of-the-art methods. Moreover, MS-SLV significantly improves multi-modal prediction, reducing the top-5 Miss Rate (${\mathrm{MR}}_{5}$) and top-10 Miss Rate (${\mathrm{MR}}_{10}$) by 26% and 33%, respectively, and lowering the Off-Road Rate (ORR) by 3%, as compared with the strongest baseline in our evaluation.

## 1. Introduction

Autonomous driving systems rely on multimodal data, encompassing both dynamic vehicle states and static map information to predict future trajectories accurately. Dynamic inputs, such as time-series vehicle states (e.g., position and velocity), are typically computed through state estimation algorithms from data collected by onboard sensors like inertial measurement units (IMUs), cameras, and radars. In contrast, static spatial information regarding lane geometry and road layout is obtained from high-definition (HD) maps. Effectively integrating these multimodal inputs is crucial for robust trajectory prediction.

A key challenge in trajectory prediction lies in effectively capturing the evolving spatial context induced by vehicle motion while maintaining prediction accuracy and diversity. Existing methods often fail to align scene semantics with agent movement, neglecting how scene features evolve over time [1]. Current latent variable models lack structured representations that disentangle individual-specific and scene-shared factors [2], leading to either overly deterministic or contextually inconsistent predictions. Additionally, trajectory forecasting and lane sequence prediction are typically treated as separate tasks [3], missing opportunities to reinforce predictive robustness through multi-task generative modeling. Addressing these limitations requires a unified framework that leverages time-indexed scene encoding, structured latent modeling, and multi-task learning to enhance prediction accuracy and diversity.

To tackle these challenges, existing work has primarily explored two aspects of multimodal trajectory prediction: enhancing input representation by unified scene encoding and employing generative models to handle multimodal outputs. Among these, recent advances have leveraged multimodal inputs, incorporating dynamic agent states and static HD map features. Existing map encoding methods typically rely on static, vectorized representations, which align well with trajectory formats [4] but overlook the evolving spatial context induced by vehicle motion. Thus, a fundamental discrepancy arises: contextual semantics evolve as the vehicle moves, while static map features remain unchanged, limiting the model’s ability to reason over context-dependent future behaviors. To generate diverse trajectory predictions, various generative models have been employed, including Generative Adversarial Networks (GANs) [5], Variational Autoencoders (VAEs) [6], and Conditional VAEs (CVAEs) [7]. Each approach exhibits notable trade-offs between prediction accuracy and trajectory diversity [8,9]. Specifically, the GAN-based methods lack a probabilistic framework, hindering their capacity to quantify uncertainty and limiting trajectory diversity; the VAE-based methods rely on overly simplistic assumptions, such as diagonal Gaussian distributions, which restricts their ability to effectively model complex and multimodal behavior patterns; and CVAE-based methods introduce conditional generation to address multimodality, so they often struggle to capture the intricate dependencies between dynamic vehicle motion and spatially evolving map features. Therefore, a unified framework that not only aligns dynamic inputs with evolving scene semantics but also incorporates structured latent space representations to generate diverse yet contextually consistent trajectories is still lacking.

In this paper, we propose a Multi-Source Input with Structured Latent Variable Model (MS-SLV), a unified framework that captures dynamic agent states and structured environmental semantics in a coherent spatio-temporal representation. Our contributions are summarized as follows:

(i) Time-aware scene encoding: We propose a time-indexed scene representation that encodes the scene layout at both present and future steps, aligning spatial semantics with the agent’s motion. This formulation captures how scene semantics evolve relative to vehicle movement, enhancing context-aware trajectory predictions, e.g., dynamic driving.

(ii) Structured latent variable modeling: We design a structured latent variable model that disentangles individual-specific and scene-shared factors through semantic partitioning and Kullback–Leibler divergence (KL) regularization. This structured latent space not only improves prediction accuracy but also balances trajectory diversity, addressing the trade-offs between deterministic and multimodal outputs.

(iii) Multi-task generative framework: We integrate structured latent modeling into a multi-task generative framework that unifies trajectory forecasting and lane sequence prediction. This formulation leverages structured latent spaces to reinforce prediction robustness while maintaining scene interpretability in complex traffic scenarios.

(iv) Extensive simulation: We evaluate the performance of our structured latent variable model on the nuScenes dataset using displacement-based metrics. Our method reduces the Average Displacement Error (ADE) by 12.37% and the Final Displacement Error (FDE) by 7.67% when considering the best-five predicted trajectories. This shows improvements in multimodal prediction accuracy.

## 2. Related Work

The state-of-the-art trajectory prediction discussed in this paper is considered from two key perspectives, i.e., multimodal input representation and generative-model-based multimodal prediction.

Effective trajectory prediction necessitates the integration of diverse contextual information, encompassing both agent dynamics and static scene features. Several studies have modeled agent–agent interactions using pooling strategies based on Long Short-Term Memory networks (LSTMs) and Convolutional Neural Networks (CNNs) [10]. Moreover, recent methods, such as GRIP++ [11], leverage Graph Neural Networks (GNNs) and Transformers to capture topological interactions. To better incorporate rich scene semantics, recent works [12,13] propose vectorized representations of HD maps—including lane centerlines and traffic signs—using points, splines, or polylines for compact encoding. Building on vectorized representations, some approaches further model map elements as graphs [14], allowing for explicit topological reasoning but at a higher computational cost. Notably, these methods enable structural alignment with agent trajectories, with maps encoded statically. Therefore, static map encodings fail to account for dynamic scene changes induced by agent movement.

Generative models play a pivotal role in multimodal trajectory prediction. To account for the inherent uncertainty in future motion, multimodal trajectory prediction aims to generate multiple plausible future trajectories for a target agent. Generative models, particularly GANs and CVAEs, have become standard approaches for this task. GAN-based methods [15] rely on adversarial training to learn the data distribution and often incorporate diversity-promoting objectives to mitigate mode averaging. However, they lack a principled probabilistic foundation, making it difficult to quantify uncertainty and making them prone to mode collapse, i.e., where only a narrow set of plausible outcomes is captured [8]. CVAEs [7] offer a more principled framework by introducing latent variables to model future uncertainty conditioned on observed context. Models such as Trajectron++ [16] enhance CVAEs by incorporating multi-agent interactions and dynamically feasible trajectory modeling. Nevertheless, most CVAE-based methods rely on simplistic priors—typically diagonal Gaussians—that limit the latent space’s expressiveness. As a result, these models struggle to balance accuracy and diversity, especially when capturing the complex dependencies between dynamic agent behavior and structured environmental context [9].

Forecasting future motion in structured environments often involves multiple correlated sub-tasks, such as trajectory prediction and lane or goal sequence prediction. Several prior works address this challenge through stage-wise architectures that first infer an agent’s intent and then generate trajectories accordingly. For instance, MultiPath++ [17] and TNT [18] follow anchor-based pipelines, while goal-driven models [19,20] adopt modular branches for destination and path estimation. While effective, these methods treat intent and trajectory prediction as separate stages or loosely coupled branches, which may limit mutual supervision and lead to semantic inconsistencies.

In summary, there is a need for expressive latent representations that effectively disentangle vehicle-specific intentions from scene-level semantics, thereby enhancing prediction accuracy and diversity while maintaining contextual coherence with the evolving scene.

## 3. Multi-Source Structured Latent Variables

In this section, we introduce the proposed MS-SLV for multitask motion forecasting. We begin by presenting the overall framework, followed by deriving the evidence lower bound (ELBO) formulation under structured latent variables.

### 3.1. Overview

**Figure 1:**
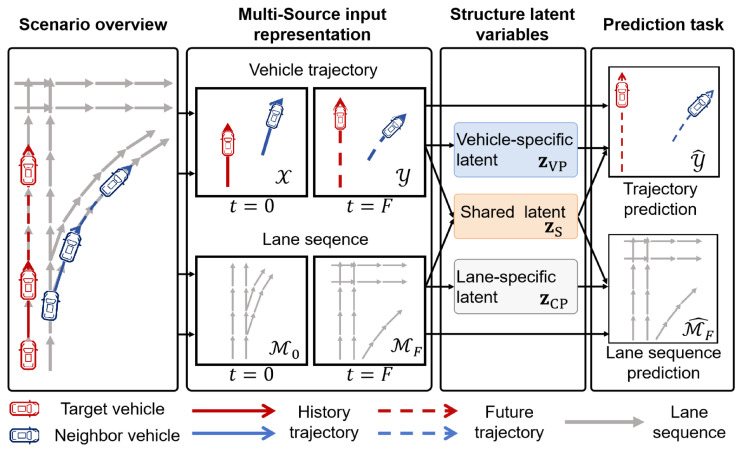
An illustration of our proposed MS-SLV.

In this paper, we consider a traffic scene where vehicle information is represented as motion trajectories and map information is represented as lane centerline sequences. As illustrated in Figure 1, vehicle trajectory information is represented as colored polylines, capturing dynamic agent states, while lane sequence information is encoded as gray polylines to reflect spatial structure. The vehicle trajectory information is represented by the observed trajectory X over the past *H* steps, which captures motion dynamics and implicitly reflects interaction. In contrast, lane sequence information is represented through spatial sequences obtained by sampling lane centerlines, providing structured scene context. To account for the contextual evolution induced by vehicle motion, lane sequences are extracted at both the current and future steps, denoted as $M_{0}$ and $M_{F}$, respectively, each centered around the target vehicle’s position.

The objective of the traffic scene is to predict the future trajectory $\hat{Y}$ of the target vehicle while concurrently forecasting the lane sequence ${\hat{M}}_{F}$. This dual-task setup is formulated as a joint conditional probability density $p(Y,M_{F}\;|\;X,M_{0})$. Our goal is to design a structured latent variable model to disentangle shared and modality-specific uncertainties, thereby capturing both scene-level context and vehicle-specific dynamics.

### 3.2. Structured Latent Variables

To address the trade-off between prediction accuracy and trajectory diversity in multimodal trajectory prediction, we introduce a structured latent variable model that decomposes trajectory uncertainty into two components: vehicle-specific latent variables $z_{V}=\left(z_{\mathrm{VP}},z_{S}\right)$ and lane-specific latent variables $z_{C}=\left(z_{\mathrm{CP}},z_{S}\right)$, as shown in Figure 1. Here, $z_{\mathrm{VP}}$ and $z_{\mathrm{CP}}$ capture vehicle- and lane-specific factors, respectively, while the shared component $z_{S}$ encodes interactions between agent behavior and contextual scene constraints. The shared latent variable $z_{S}$ serves to align vehicle behavior with environmental context, such as lane-following or deceleration near ramps, thereby promoting consistency across modalities. To prevent redundancy, $z_{\mathrm{VP}}$ and $z_{\mathrm{CP}}$ are modeled as statistically independent components, while $z_{S}$ bridges the two modalities to ensure coherent trajectory predictions.

To further encourage latent disentanglement and mitigate mode collapse, we incorporate lane sequence prediction as an auxiliary task. This complementary supervision guides the learning of modality-specific and shared representations, effectively structuring the latent space to capture both agent-specific intentions and scene-level semantics.

### 3.3. ELBO with Structured Latent Variables

To model $p\left(Y,M_{F}\;|\;X,M_{0}\right)$, we propose a CVAE-based framework with a modified ELBO that explicitly reflects the structured latent space. The modified ELBO with structure latent variables is

(1) $$\begin{array}{cc} \;\;\;\log\left[p\left(Y,M_{F}\;|\;X,M_{0}\right)\right]\;\;\geq & E_{q\left(z_{V},z_{C}|X,M_{0},Y,M_{F}\right)}\;\left[\log p\left(Y,M_{F}\;|\;z_{V},z_{C},X,M_{0}\right)\right]\\ \; & \;\;\;\;\;\;\;\;\;\;\;\;\;-\mathrm{KL}\left[q\left(z_{V},z_{C}|X,M_{0},Y,M_{F}\right)\|\;p\left(z_{V},z_{C}|X,M_{0}\right)\right]. \end{array}$$

Under the assumption of conditional independence between the two prediction targets, we decouple trajectory and lane outputs given their corresponding latent variables and observed inputs. We further factorize the ELBO into two modality-specific components and augment the latent space with a shared variable $z_{S}$, capturing shared scene-level uncertainty, and decompose the bound as

(2) $$\begin{array}{cc} \log\left[p\left(Y,M_{F}|X,M_{0}\right)\right]\geq & E_{q\left(z_{\mathrm{VP}},z_{S}|X,M_{0},Y,M_{F}\right)}\left[\log p\left(Y|z_{\mathrm{VP}},z_{S},X,M_{0}\right)\right]\\ \; & -\mathrm{KL}\left[q\left(z_{\mathrm{VP}},z_{S}|X,M_{0},Y,M_{F}\right)\|\;p\left(z_{\mathrm{VP}},z_{S}|X,M_{0}\right)\right]\\ \; & +E_{q\left(z_{\mathrm{CP}},z_{S}|X,M_{0},Y,M_{F}\right)}\left[\log p\left(M_{F}|z_{\mathrm{CP}},z_{S},X,M_{0}\right)\right]\\ \; & -\mathrm{KL}\left[q\left(z_{\mathrm{CP}},z_{S}|X,M_{0},Y,M_{F}\right)\|\;p\left(z_{\mathrm{CP}},z_{S}|X,M_{0}\right)\right]. \end{array}$$

This decomposition supports a multi-task learning paradigm, where trajectory and lane sequence prediction are modeled as parallel tasks with disentangled uncertainty sources. To further disentangle latent variables, we introduce Total Correlation (TC) regularization [21] to penalize dependencies between individual-specific $\left(z_{\mathrm{VP}},z_{\mathrm{CP}}\right)$ and shared $z_{S}$ components. Intuitively, TC encourages independence among different latent dimensions, so that each captures a distinct factor of variation, such as individual motion intent or shared contextual information. As shown in Equation (3), the training objective combines (i) reconstruction losses for vehicle and lane tasks $\left(L_{V},L_{C}\right)$; (ii) TC terms $\left({\mathrm{TC}}_{V},{\mathrm{TC}}_{C}\right)$ to reduce latent entanglement; and (iii) dimension-wise KL divergence (DW-KL) $\left({\mathrm{DW}}_{V},{\mathrm{DW}}_{C}\right)$ to align each latent unit with its priors. The DW-KL term penalizes collapsed or inactive latent units by ensuring that each dimension contributes meaningful information. Together, TC and DW-KL promote a more interpretable and robust latent space for multimodal prediction. The detailed derivation of the ELBO for structured latent variables is given in Appendix A.

(3) $$\begin{array}{cc} \log p\left(Y,M_{F}|X,M_{0}\right)\geq & \underset{L_{V}}{\underset{︸}{E_{q(z_{\mathrm{VP}},z_{S})}\left[\log p\left(Y|z_{\mathrm{VP}},z_{S},X,M_{0}\right)\right]}}\\ & +\underset{L_{C}}{\underset{︸}{E_{q(z_{\mathrm{CP}},z_{S})}\left[\log p\left(M_{F}|z_{\mathrm{CP}},z_{S},M_{0}\right)\right]}}\\ \; & -\underset{{\mathrm{TC}}_{V}}{\underset{︸}{\mathrm{KL}\left[q\left(z_{\mathrm{VP}},z_{S}\right)\|\;q\left(z_{\mathrm{VP}}\right)q\left(z_{S}\right)\right]}}-\underset{{\mathrm{TC}}_{C}}{\underset{︸}{\mathrm{KL}\left[q\left(z_{\mathrm{CP}},z_{S}\right)\|\;q\left(z_{\mathrm{CP}}\right)q\left(z_{S}\right)\right]}}\\ & -\underset{{\mathrm{DW}}_{V}}{\underset{︸}{\mathrm{KL}\left[q\left(z_{\mathrm{VP}}\right)\|\;p\left(z_{\mathrm{VP}}|X\right)\right]+\mathrm{KL}\left[q\left(z_{S}\right)\|\;p\left(z_{S}\right)\right]}}\\ \; & -\underset{{\mathrm{DW}}_{C}}{\underset{︸}{\mathrm{KL}\left[q\left(z_{\mathrm{CP}}\right)\|\;p\left(z_{\mathrm{CP}}|M_{0}\right)\right]+\mathrm{KL}\left[q\left(z_{S}\right)\|\;p\left(z_{S}\right)\right]}}. \end{array}$$

## 4. MS-SLV Workflow

**Figure 2:**
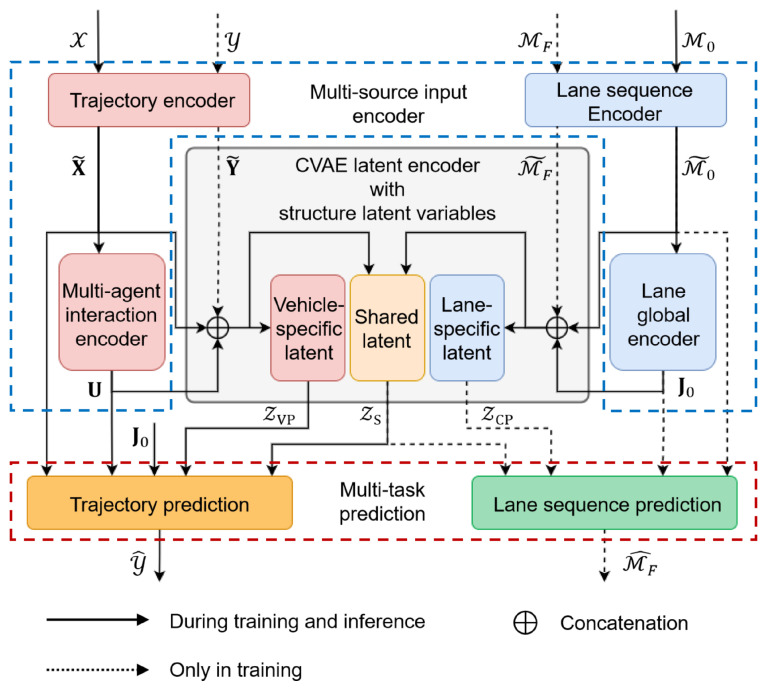
Architecture overview of MS-SLV.

We begin this section by introducing the input representations. As illustrated in Figure 2, MS-SLV consists of three main components: a multi-source input encoder, a structured latent variable encoder, and a multitask prediction decoder.

### 4.1. Input Representation

In the proposed framework, each vehicle *n* at the current step t=0 is represented by a $D_{V}$-dimensional state vector $s_{n}^{t}$, capturing its position, velocity, and heading. The historical trajectory over past *H* steps is denoted as $X_{n}=\left(s_{n}^{-H+1},\ldots ,s_{n}^{0}\right)$, while the future trajectory over *F* steps is defined as $Y_{n}=\left(s_{n}^{1},\ldots ,s_{n}^{F}\right)$. For a scene containing *N* vehicles, the input and target trajectories are expressed as $X=\left(X_{1},\ldots ,X_{N}\right)\in R^{N\times H\times D_{V}}$ and $Y=\left(Y_{1},\ldots ,Y_{N}\right)\in R^{N\times F\times D_{V}}$, respectively. To capture spatial context, we extract the *M* nearest lane centerline sequences for each vehicle within a predefined region of interest. Importantly, these sequences are sampled at both t=0 and the predicted step t=F, resulting in $M_{0}$ and $M_{F}$. This dual temporal sampling strategy underscores the evolving relevance of static lane features as the vehicle progresses through the scene. If fewer than *M* lane sequences are available, zero-padding is applied to maintain consistent tensor dimensions. Each lane sequence consists of *L* centerline vectors, each with $D_{C}$ attributes, including geometric and semantic information. Thus, for each vehicle *n*, the lane representation at step *t* is expressed as $M_{n,t}\in R^{M\times L\times D_{C}}$, and the complete lane input of *N* vehicles is $M_{t}=\left(M_{1,t},\ldots ,M_{N,t}\right)\in R^{N\times M\times L\times D_{C}}$.

### 4.2. Multi-Source Input Encoder

**Figure 3:**
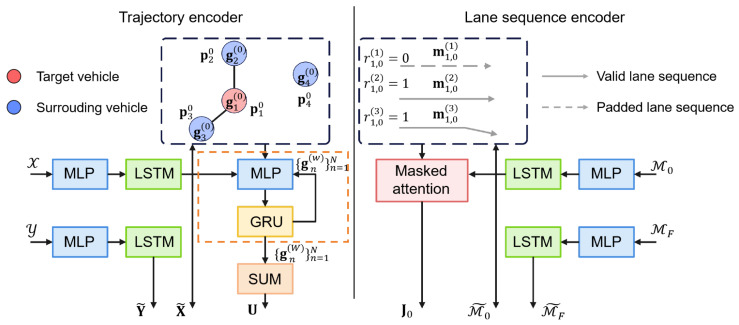
Multi-source input encoder of MS-SLV.

To capture both dynamic agent interactions and static road semantics, we propose a two-branch encoder consisting of a trajectory encoder and a lane sequence encoder, as illustrated in Figure 3.

#### 4.2.1. Trajectory Encoder

To capture both individual motion patterns and inter-agent interactions, the model adopts a two-stage encoding scheme. As illustrated in the left part of Figure 3, it first uses LSTM networks to encode temporal trajectories, followed by a GNN that models spatial interactions through message passing over a proximity-based graph.

The motion history and future trajectory of each vehicle are represented as temporal sequences of state vectors. At each step, an Multi-Layer Perceptron (MLP) encodes the raw state information $s_{n}^{t}\in R^{D_{V}}$ into a latent feature space of dimension $D_{1}$. For each sequence, the final hidden state of the corresponding LSTM is extracted as a compact representation of the entire sequence. This process yields the encoded matrices $\tilde{X}\in R^{N\times D_{1}}$ and $\tilde{Y}\in R^{N\times D_{1}}$. Specifically, ${\tilde{x}}_{n}\in R^{D_{1}}$ denotes the historical trajectory embedding of the *n*-th vehicle.

To model inter-vehicle interactions, we construct a proximity-based interaction graph where each vehicle is treated as a node. Vehicle *j* is considered a neighbor of vehicle *n* if the Euclidean distance between their positions at the initial time step satisfies $∥p_{n}^{0}-p_{j}^{0}{∥}_{2}\leq \rho_{V}$, where $p_{n}^{0}$ and $p_{j}^{0}$ denote the positions of vehicles *n* and *j* at time step t=0, respectively, and $\rho_{V}$ is a predefined distance threshold. The neighbor set of vehicle *n* is thus defined as $S_{n}=\left\{j\;|\;∥p_{n}^{0}-p_{j}^{0}{∥}_{2}\leq \rho_{V}\right\}$. The node state $g_{n}^{\left(0\right)}$ is initialized with its historical trajectory embedding ${\tilde{x}}_{n}$ and subsequently updated through *W* iterations of message passing. For each node pair (n,j) at iteration *w*, the message vector $a_{n,j}^{\left(w\right)}$ is computed by applying an MLP to the concatenation of the relative position and both node features:

(4) $$a_{n,j}^{\left(w\right)}=\mathrm{MLP}\left(\left[p_{n}^{0}-p_{j}^{0}\;∥\;g_{n}^{\left(0\right)}\;∥\;g_{j}^{\left(0\right)}\right]\right).$$

Here, [·∥·] denotes vector concatenation. Messages from neighbors are aggregated via summation and used to update node features through a GRU-based message passing mechanism:

(5) $$g_{n}^{\left(w\right)}=\mathrm{GRU}\left(\sum_{j\in S_{n}}a_{n,j}^{\left(w\right)},\;g_{n}^{\left(w-1\right)}\right).$$

Finally, the interaction embedding for vehicle *n* is computed by pooling the features of its spatial neighbors in the final layer:

(6) $$u_{n}=\sum_{j\in S_{n}}g_{j}^{\left(W\right)},$$

resulting in the interaction representation matrix $U\in R^{N\times D_{1}}$ for all vehicles.

#### 4.2.2. Lane Sequence Encoder

To incorporate spatial context from road topology, the model encodes lane segments based on their geometric and semantic features. An MLP-LSTM pipeline processes these centerline sequences, followed by masked attention to derive a lane-aware context for each vehicle. This process is outlined in the right part of Figure 3.

Each lane segment is represented as a sequence of centerline points enriched with geometric and semantic attributes. These sequences are encoded through an MLP followed by an LSTM, producing a feature vector for each lane at both time steps t=0 and t=F. The resulting feature tensors are ${\tilde{M}}_{0}\in R^{N\times M\times D_{2}}$ and ${\tilde{M}}_{F}\in R^{N\times M\times D_{2}}$. To effectively capture the spatial context around each vehicle, we compute attention weights using a masked attention mechanism among all lane embeddings at time t=0. Given *M* lane features ${\tilde{m}}_{n,0}^{\left(v\right)}\in R^{D_{2}}$, the attention score between lanes *u* and *v* is computed as $\kappa_{uv}={\tilde{m}}_{n,0}^{\left(u\right)}\cdot {\tilde{m}}_{n,0}^{(v)T}/\sqrt{D_{2}}$, and the normalized attention weight is given by $\alpha_{uv}=\exp\left(\kappa_{uv}\right)/\sum_{v^{\prime }=1}^{M}\exp\left(\kappa_{uv^{\prime }}\right).$ A binary mask $r_{n,0}^{\left(v\right)}\in \left\{0,1\right\}$ is applied to exclude padded inputs. The global lane-aware feature for vehicle *n* is

(7) $$j_{n,0}=\sum_{u=1}^{M}\sum_{v=1}^{M}\alpha_{uv}\cdot r_{n,0}^{\left(v\right)}\cdot {\tilde{m}}_{n,0}^{\left(v\right)}.$$

Stacking the outputs for all vehicles yields the global context feature matrix $J_{0}\in R^{N\times D_{2}}$.

### 4.3. Latent Variable Encoder

To capture uncertainty and disentangle vehicle-specific and lane-specific factors, the introduced structured latent variables $z_{\mathrm{VP}}$, $z_{\mathrm{CP}}$, and $z_{S}$ are modeled as

(8) $$\begin{array}{cc} q(z_{\mathrm{VP}}|X,Y) & =N\left(\mu_{\mathrm{VP}},\sigma_{\mathrm{VP}}\right), \end{array}$$

(9) $$\begin{array}{cc} q(z_{\mathrm{CP}}|M_{0},M_{F}) & =N\left(\mu_{\mathrm{CP}},\sigma_{\mathrm{CP}}\right), \end{array}$$

(10) $$\begin{array}{cc} q(z_{S}|X,M_{0},Y,M_{F}) & =N\left(\mu_{S},\sigma_{S}\right), \end{array}$$

where the normal distributions $N\left(\mu_{\mathrm{VP}},\sigma_{\mathrm{VP}}\right)$, $N\left(\mu_{\mathrm{CP}},\sigma_{\mathrm{CP}}\right)$, and $N\left(\mu_{S},\sigma_{S}\right)$ correspond to the vehicle-specific, lane-specific, and shared latent variables, with each pair $\left(\mu_{\cdot },\sigma_{\cdot }\right)$ denoting the mean and standard deviation of the corresponding Gaussian distribution.

**Figure 4:**
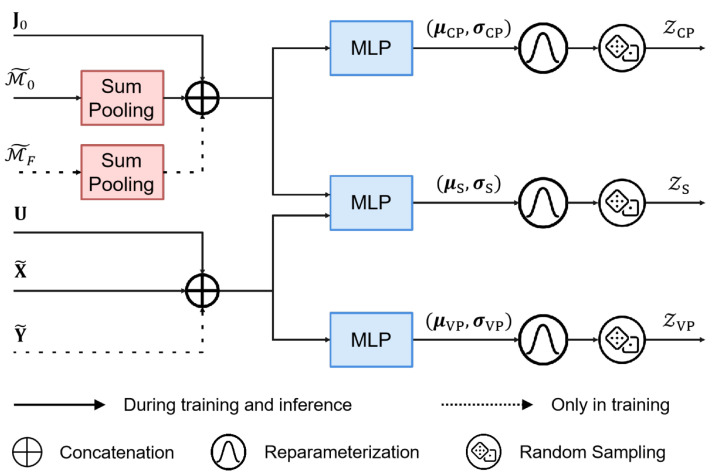
Latent variable encoder of MS-SLV.

Each posterior distribution is parameterized by an MLP tailored to specific input features, as illustrated in Figure 4. The vehicle-specific latent variable $z_{\mathrm{VP}}\in R^{D_{3}}$ is conditioned on the encoded past and future trajectories $\tilde{X}$ and $\tilde{Y}$, along with the interaction-aware feature U. For the lane-specific latent variable $z_{\mathrm{CP}}\in R^{D_{4}}$, lane encodings ${\tilde{M}}_{0}$ and ${\tilde{M}}_{F}$ are aggregated using sum pooling along the lane dimension and combined with the global context vector $J_{0}$. The shared latent variable $z_{S}\in R^{D_{5}}$ fuses vehicle and lane inputs to capture modality-independent factors. During training, latent variables are sampled using the reparameterization trick; during inference, they are drawn from priors with the same architecture, excluding future information $\tilde{Y}$ and ${\tilde{M}}_{F}$. The resulting latent tensors $Z_{\mathrm{VP}}\in R^{N\times K\times D_{3}}$, $Z_{\mathrm{CP}}\in R^{N\times K\times D_{4}}$, and $Z_{S}\in R^{N\times K\times D_{5}}$, generated via *K* random samplings, serve as disentangled factors that guide both trajectory and lane sequence predictions, effectively capturing vehicle-specific, lane-specific, and shared uncertainties.

### 4.4. Multi-Task Prediction

We jointly predict future trajectories and corresponding lane-level motion intentions, introduced as follows.

#### 4.4.1. Trajectory Prediction

**Figure 5:**
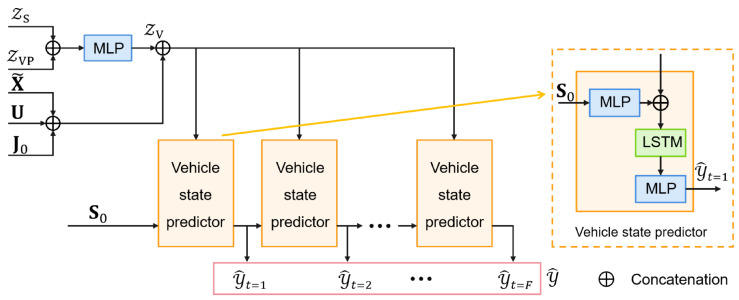
Trajectory prediction component of MS-SLV.

The structure of trajectory prediction component is shown in Figure 5. To generate *K* multimodal trajectories for each vehicle, the vehicle-specific latent $Z_{\mathrm{VP}}$ and shared latent $Z_{S}$ are fused through an MLP to form the complete latent feature $Z_{V}\in R^{N\times K\times (D_{3}+D_{5})}$. This feature is combined with the historical encoding $\tilde{X}$, interaction-aware feature U, and global lane context $J_{0}$ to construct a unified context vector for decoding. Starting from the current state $S^{0}$, an LSTM decoder autoregressively generates *K* trajectory hypotheses $\hat{Y}\in R^{N\times K\times F\times D_{V}}$, effectively capturing multiple plausible futures under varying contextual conditions.

#### 4.4.2. Lane Sequence Prediction

**Figure 6:**
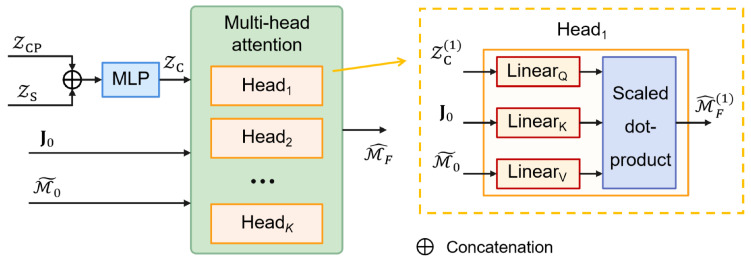
Lane sequence prediction component of MS-SLV.

The structure of the lane sequence prediction component is illustrated in Figure 6. To promote effective disentanglement and stable learning of latent variables, the lane sequence prediction task is introduced as an auxiliary supervision signal.

To model lane-level motion intent, the lane-specific latent $Z_{\mathrm{CP}}$ and shared latent $Z_{S}$ are fused through an MLP to form the composite latent vector $Z_{C}$. This vector interacts with candidate lane features via a multi-head attention (MHA) module, where the fused vehicle-lane context serves as the query and lane features ${\tilde{M}}_{0}$ as the key and value, modulated by the global lane context $J_{0}$. For each candidate lane *m*, the model generates *K* future lane sequences of length *L*, forming ${\hat{M}}_{n,F}\in R^{M\times K\times L\times D_{C}}$. Aggregating over all vehicles, the predicted lane sequences are structured as ${\hat{M}}_{F}\in R^{N\times M\times K\times L\times D_{C}}$, enabling to jointly reason over vehicle intent and scene topology.

This auxiliary task encourages the lane-specific latent $Z_{\mathrm{CP}}$ to specialize in encoding road environment features, while the vehicle-specific latent $Z_{\mathrm{VP}}$ focuses on personalized vehicle dynamics. Meanwhile, the shared latent $Z_{S}$ effectively captures common features, preventing mode collapse and enhancing the interpretability of the latent space. The beneficial role of this auxiliary task is further supported by the experimental results presented in Section 5.3.

### 4.5. Objective Function

Within the CVAE framework, trajectory and lane sequence prediction are formulated as reconstruction tasks, with Gaussian observation noise modeled through Mean Squared Error (MSE). For vehicle trajectories, the reconstruction loss is defined as the average MSE across all vehicles, prediction steps, and *K* samples, capturing the discrepancy between predicted and ground-truth trajectories:

(11) $$L_{V}^{\prime }=\frac{1}{NKF}\sum_{n=1}^{N}\sum_{k=1}^{K}\sum_{t=1}^{F}{∥{\hat{Y}}_{n}^{\left(k\right)}-Y_{n}∥}_{2}^{2}.$$

To account for mode coverage and mitigate the impact of outliers in lane sequence prediction, we adopt a minimum-MSE loss over *K* hypotheses, following the multiple-choice learning framework [22]:

$$L_{C}^{\prime }\;=\;\frac{1}{N}\;\sum_{n=1}^{N}\min_{k}\;\left\{\;\frac{1}{ML}\;\sum_{m=1}^{M}\;\sum_{l=1}^{L}\;{∥{\hat{M}}_{n,F}^{\left(m,k\right)}\;-\;M_{n,F}^{\left(m\right)}∥}_{2}^{2}\;\right\}.$$

The overall objective, as defined in Equation (3), integrates the reconstruction terms with a KL divergence regularization, incorporating both TC and DW-KL components:

$$\begin{array}{c} \;\;\;\;\;\;\;\;\;\;\;\;\;L\;\;=\;\;L_{V}^{\prime }\;+\;\lambda_{C}L_{C}^{\prime }\;-\;\lambda_{\mathrm{KL}}\left[\beta_{1}\left({\mathrm{TC}}_{V}\;+\;{\mathrm{TC}}_{C}\right)\;+\;\beta_{2}\left({\mathrm{DW}}_{V}\;+\;{\mathrm{DW}}_{C}\right)\right]. \end{array}$$

Here, $\lambda_{C}$ balances the importance of lane sequence reconstruction, $\lambda_{\mathrm{KL}}$ scales the KL term, and $\beta_{1}$ and $\beta_{2}$ control the contributions of TC and DW-KL terms, respectively.

## 5. Experiments

This section begins with the experimental settings, followed by overall performance comparison, ablation studies, and qualitative visualization.

### 5.1. Experiment Settings

We evaluate the proposed framework using the nuScenes dataset [23], which comprises 1000 driving scenes with high-definition maps and annotated 3D bounding boxes. The input data consists of 2 s of historical trajectories sampled at 2 Hz, utilized to predict the 6 s of vehicle motion. For each target vehicle, we consider surrounding agents within a 50 m radius ($\rho_{V}=50$) and extract lane features from a $50\times 50\;m^{2}$ region centered on the target vehicle. The main hyperparameters of the model are set as follows: the input feature dimensions for vehicles and lanes are $D_{V}=4$ and $D_{C}=5$, respectively; the vehicle and lane embedding dimensions are $D_{1}=8$ and $D_{2}=32$; the dimensions of the personalized latent variables for vehicles and lanes are $D_{3}=4$ and $D_{4}=4$; and the dimension of the shared latent variable is $D_{5}=8$. The KL divergence weight is gradually increased from an initial value $\lambda_{0}$ to $\lambda_{1}$ following a sigmoidal annealing schedule:

(12) $$\lambda_{\mathrm{KLD}}\left(E\right)=\lambda_{0}+\left(\lambda_{1}-\lambda_{0}\right)/\left(1+e^{-\kappa \cdot \left(E-E_{c}\right)}\right),$$

where *E* is the training epoch, κ controls the steepness, and $E_{c}$ is the center of the curve.

We evaluate performance using ${\mathrm{ADE}}_{K}$ and ${\mathrm{FDE}}_{K}$ [15], which measure the average and final displacement errors between the ground truth and the best of *K* predicted trajectories per sample:

(13) $$\begin{array}{cc} {\mathrm{ADE}}_{K} & =\min_{k}\frac{1}{NT}\sum_{n=1}^{N}\sum_{t=1}^{T}{∥{\hat{Y}}_{n,t}^{\left(k\right)}-Y_{n,t}∥}_{2},\\ {\mathrm{FDE}}_{K} & =\min_{k}\frac{1}{N}\sum_{n=1}^{N}{∥{\hat{Y}}_{n,F}^{\left(k\right)}-Y_{n,F}∥}_{2}. \end{array}$$

All displacement-based metrics (${\mathrm{ADE}}_{K}$, ${\mathrm{FDE}}_{K}$) are reported in meters throughout the paper. In addition to evaluating the diversity and physical feasibility of predictions, we include the Miss Rate at top-*K* (${\mathrm{MR}}_{K}$) and Off-Road Rate (ORR). The Miss Rate measures the fraction of agents for which none of the predicted trajectories fall within 2 m of the ground truth at the final time step. This metric reflects the model’s ability to generate at least one accurate prediction among *K* candidates:

(14) $$\begin{array}{c} {\mathrm{MR}}_{K}=\frac{1}{N}\sum_{n=1}^{N}I\left[\min_{k}\left({∥{\hat{Y}}_{n,F}^{\left(k\right)}-Y_{n,F}∥}_{2}\right)>2\right], \end{array}$$

where the indicator function $I\left[\cdot \right]$ is used to count the number of missed predictions, equal to 1 if the minimum distance between any predicted trajectory and the ground truth exceeds the threshold.

The Off-Road Rate quantifies the proportion of predicted trajectories that leave the drivable area as defined by the map. This metric captures whether predictions are physically realistic and conform to road constraints:

(15) $$\mathrm{ORR}=\frac{\mathrm{Number}\;\mathrm{of}\;\mathrm{off}\text{-}\mathrm{road}\;\mathrm{trajectories}}{\mathrm{Total}\;\mathrm{number}\;\mathrm{of}\;\mathrm{trajectories}}.$$

All experiments are conducted on an Ubuntu 22.04.4 system equipped with an Intel 16-core CPU (2.3 GHz), 128 GB of RAM, and a single NVIDIA RTX 3080 Ti GPU.

### 5.2. Comparison Experiments

We evaluate MS-SLV against five state-of-the-art baselines, i.e., Constant Velocity and Heading Angle (CVHA) [24] (physics-based model), Physics Oracle [24] (heuristic approach), MTP [25], MultiPath [26], and AgentFormer [2]. Specifically, CVHA assumes constant speed and heading throughout the prediction horizon and Physics Oracle selects the best outcome from four physics-driven variants based on velocity, acceleration, and angular data. MTP employs a CNN-based architecture to generate a fixed number of trajectories, optimized through regression and classification losses. MultiPath [26] learns anchor trajectories and regresses residuals relative to these anchors to capture plausible future motions. AgentFormer [2] combines a CVAE with Transformers to model multimodal distributions, employing a staged training approach to first learn trajectory dependencies and then refine latent distributions. To assess the robustness of performance differences, the results of the proposed method are averaged over five independent runs with different random seeds. We report the mean and standard deviation of ${\mathrm{ADE}}_{K}$ and ${\mathrm{FDE}}_{K}$. The symbol “↓” indicates that a lower value represents better performance.

Quantitative results for predicted trajectories K=1, 5, 10 are summarized in Table 1, evaluated using ${\mathrm{ADE}}_{K}$ and ${\mathrm{FDE}}_{K}$ defined in (13). At K=1, the MS-SLV achieves the lowest ${\mathrm{ADE}}_{1}$ and ${\mathrm{FDE}}_{1}$, indicating its effectiveness in modeling dominant motion patterns such as routine driving and turning behaviors. At K=5, MS-SLV still outperforms all baselines, demonstrating a substantial reduction in ${\mathrm{FDE}}_{5}$ compared to AgentFormer and MultiPath, reflecting its ability to generate diverse yet contextually aligned trajectories. As for K=10, MS-SLV maintains competitive performance, achieving the lowest ${\mathrm{ADE}}_{10}$ and a comparable ${\mathrm{FDE}}_{10}$, highlighting its robustness in multimodal prediction across varying levels of trajectory diversity.

Table 2 presents the quantitative comparison of different methods on the nuScenes dataset. The proposed method, MS-SLV, achieves the best performance in terms of both prediction accuracy and safety. Specifically, MS-SLV reduces the ${\mathrm{MR}}_{5}$ to 48% and ${\mathrm{MR}}_{10}$ to 40%, outperforming both MTP (74% and 67%) and MultiPath (78% and 76%). This indicates that our method better captures future trajectories within a limited number of prediction modes. In addition, the ORR is reduced to 9%, showing that MS-SLV generates more feasible and road-compliant predictions. The small standard deviations further demonstrate the consistency of our results across training runs.

### 5.3. Ablation Study on Modules

First, to assess the impact of lane-related components, we conduct an ablation study with two experimental settings on the nuScenes dataset. In the first setting (Group 1), only vehicle trajectories are used as input, with all lane features excluded. In the second setting (Group 2), lane features are included, but the auxiliary lane sequence prediction task is disabled. As shown in Table 3, the exclusion of lane inputs in Group 1 leads to a marked decline in performance, particularly in ${\mathrm{FDE}}_{K}$, highlighting the critical role of scene context in long-term endpoint prediction. Higher ${\mathrm{MR}}_{K}$ and ORR values compared to the full model indicate more frequent prediction failures and less trajectory diversity. Comparing Group 2 with the complete model further underscores the benefits of the auxiliary lane sequence prediction task, which provides additional supervision for the lane-related latent variable $z_{C}$. The inclusion of this auxiliary task not only improves overall accuracy but also stabilizes training by mitigating overfitting through multi-task regularization.

Secondly, we analyze how map input availability affects model performance. The model encodes the sequence of surrounding lanes associated with the target agent at each time step, explicitly incorporating road structure and environmental constraints into trajectory and interaction representations. This design makes the model inherently reliant on complete map information. As shown in Table 4, with full map availability (100%), the model achieves optimal accuracy by effectively modeling agent–environment interactions. In contrast, at 75% and 50% availability, multimodal prediction errors (${\mathrm{ADE}}_{K}$ and ${\mathrm{FDE}}_{K}$) increase notably across different values of *K*, indicating that missing map context impairs the model’s ability to capture dominant motion patterns and generate diverse, plausible futures. Notably, ${\mathrm{FDE}}_{K}$ shows a larger relative increase than ${\mathrm{ADE}}_{K}$, reflecting a greater sensitivity in long-horizon prediction to incomplete map inputs. These results underscore the importance of high-quality map data for reliable forecasting.

### 5.4. Ablation Analysis of Latent Variables

We investigate the design of the structured latent variables from composition and dimensionality perspectives.

#### 5.4.1. Latent Composition

To assess the contribution of each latent component, we conduct ablation studies by selectively enabling $z_{\mathrm{VP}}$ (vehicle-specific), $z_{\mathrm{CP}}$ (lane-specific), and $z_{S}$ (shared) latent variables. The results illustrated in Table 5 reveal that using only $z_{\mathrm{VP}}$ overlooks scene context, using only $z_{\mathrm{CP}}$ fails to capture vehicle-specific details, and using only $z_{S}$ lacks specificity. Incorporating all components improves performance but introduces redundancy, while the optimal configuration is achieved with the combination of $z_{\mathrm{VP}}$ and $z_{S}$, showing the effectiveness of disentangled representations. Notably, the weaker performance of the $z_{\mathrm{CP}}$-only configuration (as shown in Table 5 and Table 6) stems from two factors: (i) lane-level features alone cannot differentiate vehicles with distinct motion intentions but similar spatial context; and (ii) lane encodings tend to be homogeneous across nearby agents, reducing the representational diversity of $z_{\mathrm{CP}}$. This leads to less personalized and sometimes mode-collapsed predictions, reflected in elevated miss rates and reduced robustness.

#### 5.4.2. Latent Dimensionality

The impact of latent dimensionality is further examined by varying the size of each latent component, as shown in Table 7. The dimensions $D_{3}$ ($z_{\mathrm{VP}}$), $D_{4}$ ($z_{\mathrm{CP}}$), and $D_{5}$ ($z_{S}$) correspond to the latent variables introduced in Section 4.3. Setting the individual-specific dimensions to four and shared dimension to eight (Group 4) achieves the best trade-off between accuracy and diversity. Reducing the dimensions constrains MS-SLV’s capacity, whereas excessively large dimensions lead to overfitting and increased noise. Expanding only $D_{4}$ (Group 5) degrades performance due to redundant lane encoding, but this can be mitigated by increasing the size of $D_{5}$ (Group 6). Further increasing both components simultaneously (Group 3) does not yield additional gains, indicating that the optimal latent dimensionality requires joint tuning across all components.

Furthermore, we observe a performance drop when the lane-specific latent $z_{\mathrm{CP}}$ becomes overly highly dimensional (e.g., Groups 5 and 6). Although $z_{\mathrm{CP}}$ is not directly used for trajectory decoding, increasing its capacity may cause the model to overfit to fine-grained lane variations—such as slight curvature or width differences—that do not generalize across scenes. This over-specialization can interfere with shared or vehicle-specific representations, leading to entangled or redundant encodings. In addition, higher-dimensional latents increase memory and compute overhead during training and inference, especially in multi-agent settings. These results highlight a key trade-off: while more expressive latents offer modeling flexibility, excessive capacity in auxiliary components like $z_{\mathrm{CP}}$ can harm generalization and efficiency. This underscores the importance of proper dimension tuning to balance flexibility and generalization.

### 5.5. Impact of Regularization Weight

We investigate the impact of regularization weight from KL term decomposition and KL weighting strategy aspects.

#### 5.5.1. KL Term Decomposition

To enhance the disentanglement of structured latent variables, we decompose the KL divergence into TC and DW-KL components, each with separate regularization terms for the three latent variables. The impact of this decomposition is examined in Table 8. The results indicate that both weighting coefficients play a crucial role in training stability and predictive performance. TC regularization appears crucial for stable training and consistent latent structuring, as evidenced by the performance with $\beta_{1}=1.0$. In contrast, DW-KL primarily contributes to improved predictive accuracy. These findings demonstrate the complementary roles of TC and DW-KL in effectively structuring the latent space.

#### 5.5.2. KL Weighting Strategy

We further investigate the impact of static versus dynamic scheduling strategies for KL weights. The dynamic schedule follows a sigmoidal curve parameterized by $\left(\lambda_{0},\lambda_{1},\kappa \right)$ under three configurations: Fast (0.1,1.0,10.0), High (0.1,2.0,5.0), and Steady (0.1,1.0,5.0). As reported in Table 9, the Steady schedule achieves the optimal balance between reconstruction fidelity and latent structure across varying sample sizes. In contrast, overly aggressive dynamics or weak static weights degrade performance, particularly when the number of predicted samples K=1 is small, highlighting the importance of calibrated KL weight scheduling in stabilizing training dynamics.

### 5.6. Computational Efficiency

To evaluate the real-time performance of the proposed MS-SLV model, we measure its per-sample inference time on an NVIDIA RTX 3080 Ti GPU. The model achieves an average inference time of 40.6 ms with a standard deviation of 3.8 ms, corresponding to a throughput of approximately 24.6 samples per second. For comparison, AgentFormer—another CVAE-based generative model for trajectory prediction—requires 99.6 ms per sample on the same hardware. This highlights the efficiency of our multi-task architecture, making it suitable for real-time deployment.

Regarding training efficiency, MS-SLV consumes around 520 MiB of GPU memory per sample during inference and requires 0.273 Giga Floating Point Operations (GFLOPs), which is lower than our reimplementation of AgentFormer (634 MiB, 3.084 GFLOPs) under the same hardware conditions. These results demonstrate that MS-SLV achieves superior runtime efficiency in both memory usage and computational complexity while remaining lightweight enough for real-time deployment.

Additionally, MS-SLV takes approximately 0.31 h per epoch on a single RTX 3080 Ti GPU and typically converges within 50 epochs. By contrast, AgentFormer requires about 0.66 h per epoch and converges within 100 epochs under identical hardware and training configurations. This demonstrates that MS-SLV achieves improved training efficiency in both per-epoch speed and total training time.

### 5.7. Qualitative Analysis and Visualization

To better understand the behavior of MS-SLV across diverse traffic scenarios, we qualitatively examine predicted trajectories in conjunction with lane geometry, surrounding vehicles, and temporal evolution.

**Figure 7:**
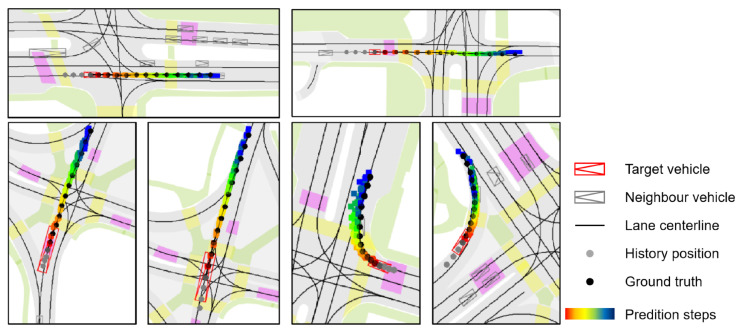
Prediction in simple driving scenarios.

**Figure 8:**
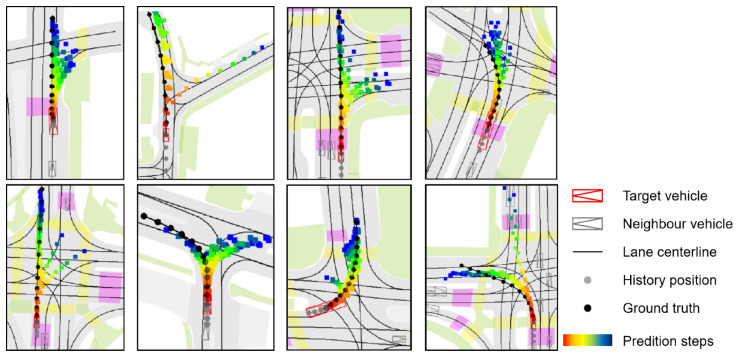
Prediction in complex driving scenarios.

**Visualization legend.** In Figure 7 and Figure 8, vehicles are shown as composite symbols combining rectangles and triangles, with triangles indicating heading direction. The red symbol marks the target vehicle; gray symbols denote surrounding vehicles. Solid black lines represent lane centerlines from HD maps. The target vehicle’s historical trajectory is shown as gray dots and its ground-truth future trajectory as black dots. Predicted future positions use color-coded dots from red to dark blue, indicating progression over time, with red corresponding to earlier time steps and blue to later time steps.

Figure 7 illustrates MS-SLV’s predictions in simple driving scenarios, such as straight driving or gentle turns. The predicted trajectories align closely with the ground truth, indicating high precision and temporal consistency. The model captures variations in vehicle speed, lane curvature, and local agent interactions, maintaining accuracy even in denser contexts. This confirms MS-SLV’s ability to model deterministic behavior patterns when the motion intent is relatively unambiguous.

Figure 8 focuses on more complex and uncertain driving situations, including intersections, merges, and multi-path zones. In these cases, the model produces multiple plausible futures, with distinct trajectory branches clearly visible through their diverging spatial paths and temporal color gradients. This reflects MS-SLV’s capacity to capture multimodal intent and leverage lane semantics to generate scene-consistent alternatives. The predicted modes respect road structure and remain feasible under dynamic constraints, highlighting the model’s robustness in uncertain environments.

Overall, these visualizations reinforce our quantitative findings: MS-SLV generates accurate predictions in low-uncertainty settings and maintains high diversity in ambiguous scenarios, supporting safe and reliable motion forecasting across varied traffic contexts.

### 5.8. Result and Discussion

We conduct comprehensive experiments on the nuScenes dataset to assess the performance, component contributions, regularization effects, and deployment practicality of the proposed MS-SLV model.

**Overall performance.** MS-SLV achieves state-of-the-art results across all core metrics on the nuScenes dataset (Table 1). It consistently outperforms five strong baselines in both accuracy (e.g., lowest ${\mathrm{ADE}}_{1}$ and ${\mathrm{FDE}}_{1}$) and diversity (notably lower ${\mathrm{MR}}_{5}$, ${\mathrm{MR}}_{10}$, and ORR). These improvements validate the effectiveness of its structured latent design and auxiliary scene supervision.

**Ablation study on model components.** We ablate lane-related components to assess the role of scene context and auxiliary supervision. Removing lane inputs or disabling the auxiliary lane sequence task (Table 3) leads to clear drops in accuracy and diversity, especially in long-horizon ${\mathrm{FDE}}_{K}$, confirming the value of spatial cues and multi-task learning for structured latent modeling. Additionally, map availability experiments (Table 4) show that performance deteriorates as lane context decreases, with larger impacts on ${\mathrm{FDE}}_{K}$. This highlights the model’s strong reliance on complete map inputs.

**Latent variable structure and regularization.** We evaluate how latent composition and regularization affect performance. Combining vehicle-specific $z_{\mathrm{VP}}$ and shared $z_{S}$ achieves the best results, validating the benefit of disentangled latent modeling. Lane-specific $z_{\mathrm{CP}}$ alone is less effective due to limited agent discrimination. Moderate latent dimensions (e.g., $D_{3}=4$, $D_{5}=8$) strike a good balance between expressiveness and generalization. Regularizing KL divergence via TC and DW-KL further improves stability and accuracy. A steady KL annealing schedule proves most robust across sampling settings, highlighting the importance of calibrated regularization in latent space learning.

**Computational efficiency and qualitative insights.** MS-SLV demonstrates real-time performance, with 40.6 ms inference time per sample, while maintaining low memory and computational cost. Compared to baseline, it offers faster inference, a reduced GPU memory footprint, and more efficient training. These results collectively validate MS-SLV’s suitability for real-time applications under hardware constraints.

In summary, the proposed MS-SLV framework achieves state-of-the-art performance through effective integration of structured latent variables, auxiliary supervision, and scene-aware reasoning. Each component—from model architecture to regularization and input encoding—contributes meaningfully to accurate, diverse, and reliable trajectory prediction.

## 6. Conclusions

To achieve both accuracy and diversity in trajectory prediction, it is essential to capture the evolving spatial context induced by vehicle motion, which depends critically on the effective fusion of multimodal inputs—including dynamic vehicle states and static HD map features. To this end, we propose MS-SLV, a unified framework that leverages time-aware scene encoding, structured latent space modeling, and multi-task generative learning. MS-SLV disentangles vehicle-specific and scene-shared factors and incorporates lane sequence prediction to improve multimodal trajectory forecasting performance. Comparison experiments on the nuScenes dataset demonstrate consistent improvements over baselines in multimodal prediction accuracy, with up to a 12.37% reduction in ADE and 7.67% in FDE, as well as 26% and 33% reductions in ${\mathrm{MR}}_{5}$ and ${\mathrm{MR}}_{10}$, respectively, and a 3% drop in ORR. These results demonstrate that MS-SLV achieves accurate, diverse, and physically plausible trajectory predictions. The model also maintains robust performance across varying numbers of predicted trajectories, highlighting its strong mode coverage. Extensive ablation studies validate the effectiveness of structural latent modeling, joint trajectory and lane sequence prediction, and the regularization terms in shaping the latent space. Furthermore, our qualitative results confirm that the proposed MS-SLV framework is capable of generating diverse and scene-compliant future trajectories. Visualizations in complex scenarios, including intersections, demonstrate its potential to capture multimodal futures consistent with the scene context, indicating robustness in challenging conditions.

While MS-SLV demonstrates strong performance, it has several limitations. The framework involves relatively high computational complexity due to latent variable sampling and lane sequence prediction. It also relies heavily on accurate HD map data, which may not always be available or up-to-date in real-world scenarios. Moreover, although evaluated on the nuScenes dataset, further assessment on diverse datasets is necessary to verify the model’s generalization capabilities. Future work may explore incorporating environmental factors such as weather conditions (e.g., rain, snow, fog) and road surface states (e.g., wet or icy surfaces), which significantly affect vehicle behavior and trajectory prediction accuracy. Our framework naturally supports the integration of these factors as additional input modalities, such as through sensor data or external environmental information, which can enhance context-aware trajectory prediction. Moreover, the model’s robustness and generalizability can be further assessed by testing it under diverse scenarios, including complex traffic patterns and adverse weather conditions, through the use of broader datasets and simulation environments. Additionally, efforts to reduce prediction latency will be essential for enabling real-time deployment in safety-critical applications. Incorporating these factors into the MS-SLV framework could improve prediction accuracy and applicability across varied driving scenarios. 

## Figures and Tables

**Table 1:** Quantitative comparison with baseline methods on the nuScenes dataset.

Method	${\mathrm{ADE}}_{1}$ (m)↓	${\mathrm{FDE}}_{1}$ (m)↓	${\mathrm{ADE}}_{5}$ (m)↓	${\mathrm{FDE}}_{5}$ (m)↓	${\mathrm{ADE}}_{10}$ (m)↓	${\mathrm{FDE}}_{10}$ (m)↓
CVHA	4.61	11.21	4.61	11.21	4.61	11.21
Physics Oracle	3.69	9.06	3.69	9.06	3.69	9.06
MTP	4.42	10.36	2.22	4.83	1.74	3.54
MultiPath	4.43	10.16	1.78	3.62	1.55	2.93
AgentFormer	4.09	9.47	1.86	3.89	1.45	**2.86**
**MS-SLV (Ours)**	**2.43 ± 0.05**	**5.81 ± 0.11**	**1.63 ± 0.04**	**3.58 ± 0.08**	**1.44 ± 0.03**	3.04 ± 0.07

**Table 2:** Evaluation of multi-modal prediction accuracy and safety on the nuScenes dataset.

Method	${\mathrm{MR}}_{5}$ (%)↓	${\mathrm{MR}}_{10}$ (%)↓	ORR (%)↓
CVHA	91	91	14
Physics Oracle	88	88	12
MTP	74	67	25
MultiPath	78	76	36
**MS-SLV (Ours)**	**48 ± 2**	**40 ± 1**	**9 ± 1**

**Table 3:** Ablation study of modules.

Methods	ADE_1_↓	FDE_1_↓	ADE_5_↓	FDE_5_↓	ADE_10_↓	FDE_10_↓	MR_5_↓	MR_10_↓	ORR↓
Group 1	3.97	9.80	2.28	5.36	1.89	4.30	78	65	25
Group 2	4.43	10.84	3.31	7.85	3.04	7.11	87	73	42
**Full Model**	**2.43**	**5.81**	**1.63**	**3.58**	**1.44**	**3.04**	**48**	**40**	**9**

**Table 4:** Ablation study of map input availability.

Avail.	ADE_1_↓	FDE_1_↓	ADE_5_↓	FDE_5_↓	ADE_10_↓	FDE_10_↓	MR_5_↓	MR_10_↓	ORR↓
100%	2.43	5.81	1.63	3.58	1.44	3.04	48	40	9
75%	3.19	7.89	2.10	4.88	1.82	4.06	64	56	18
50%	3.22	7.91	2.12	4.96	1.84	4.20	68	60	21

**Table 5:** Ablation study of latent composition.

Methods	$z_{\mathrm{VP}}$	$z_{\mathrm{CP}}$	$z_{S}$	${\mathrm{ADE}}_{1}$↓	${\mathrm{FDE}}_{1}$↓	${\mathrm{ADE}}_{5}$↓	${\mathrm{FDE}}_{5}$↓	${\mathrm{ADE}}_{10}$↓	${\mathrm{FDE}}_{10}$↓
Group 1	✓	×	×	2.89	8.14	1.84	4.97	1.62	4.23
Group 2	×	✓	×	4.57	11.03	3.39	8.19	3.05	7.29
Group 3	×	×	✓	4.38	10.83	2.64	6.29	2.21	5.13
Group 4	×	✓	✓	3.39	8.44	2.17	5.14	1.88	4.29
Group 5	✓	✓	✓	2.54	6.05	1.71	3.81	1.51	3.25
**MS-SLV (Ours)**	✓	×	✓	**2.43**	**5.81**	**1.63**	**3.58**	**1.44**	**3.04**

**Table 6:** Complementary metrics for latent composition ablation.

Methods	$z_{\mathrm{VP}}$	$z_{\mathrm{CP}}$	$z_{S}$	MR_5_↓	MR_10_↓	ORR↓
Group 1	✓	×	×	61	52	19
Group 2	×	✓	×	87	73	41
Group 3	×	×	✓	72	63	30
Group 4	×	✓	✓	56	47	16
Group 5	✓	✓	✓	50	42	11
**MS-SLV (Ours)**	✓	×	✓	**48**	**40**	**9**

**Table 7:** Ablation study on latent variable dimensionality.

Methods	Dimension			K=1		K=5		K=10	
	$D_{3}$	$D_{4}$	$D_{5}$	${\mathrm{ADE}}_{1}$ **↓**	${\mathrm{FDE}}_{1}$ **↓**	${\mathrm{ADE}}_{5}$ **↓**	${\mathrm{FDE}}_{5}$ **↓**	${\mathrm{ADE}}_{10}$ **↓**	${\mathrm{FDE}}_{10}$ **↓**
Group 1	2	2	2	2.61	6.22	1.78	3.95	1.59	3.40
Group 2	4	4	4	2.49	5.85	1.68	3.64	1.49	3.12
Group 3	8	8	8	2.69	6.44	1.70	3.79	1.49	3.19
Group 4	4	4	8	**2.43**	**5.81**	**1.63**	**3.58**	**1.44**	**3.04**
Group 5	4	8	4	3.14	7.65	2.13	4.89	1.90	4.24
Group 6	4	8	8	2.68	6.53	1.90	4.37	1.71	3.82

**Table 8:** Ablation of KL weights $\beta_{1}$ (TC) and $\beta_{2}$ (DW-KL).

β		K=1		K=5		K=10	
$\beta_{1}$	$\beta_{2}$	${\mathrm{ADE}}_{1}$ **↓**	${\mathrm{FDE}}_{1}$ **↓**	${\mathrm{ADE}}_{5}$ **↓**	${\mathrm{FDE}}_{5}$ **↓**	${\mathrm{ADE}}_{10}$ **↓**	${\mathrm{FDE}}_{10}$ **↓**
1.0	0.0	3.09	7.44	2.01	4.54	1.77	3.89
1.0	1.0	**2.43**	**5.81**	**1.63**	**3.58**	**1.44**	**3.04**

**Table 9:** Comparison of dynamic and static KL weighting strategies.

Type	Strategy	K=1		K=5		K=10	
		**ADE_1_↓**	**FDE_1_↓**	**ADE_5_↓**	**FDE_5_↓**	**ADE_10_↓**	**FDE_10_↓**
Dynamic	Fast	2.60	6.27	1.71	3.82	1.51	3.24
	High	2.92	7.13	1.91	4.37	1.68	3.74
	**Steady**	**2.43**	**5.81**	**1.63**	**3.58**	**1.44**	**3.04**
Static	1.0	4.53	11.02	3.34	8.13	2.98	7.20
	2.0	**2.51**	**5.82**	**1.85**	**4.05**	**1.68**	**3.58**

## Data Availability

The data used in this study are publicly available as part of the nuScenes dataset, which can be accessed at https://www.nuscenes.org (accessed on 10 June 2025).

## Abbreviations

The following abbreviations are used in this manuscript:

IMUs	Inertial Measurement Units
HD	High Definition
MS-SLV	Multi-Source Input with Structured Latent Variable Model
CNNs	Convolutional Neural Networks
GNNs	Graph Neural Networks
LSTMs	Long Short-Term Memory networks
GANs	Generative Adversarial Networks
VAEs	Variational Autoencoders
CVAEs	Conditional Variational Autoencoders
MLP	Multi-Layer Perceptron
GRUs	Gated Recurrent Units
ELBO	Evidence Lower Bound
KL	Kullback–Leibler Divergence
ADE	Average Displacement Error
FDE	Final Displacement Error
MR	Miss Rate
ORR	Off-Road Rate
TC	Total Correlation
GFLOPs	Giga Floating Point Operations
DW-KL	Dimension-Wise KL Divergence

## Appendix A. Derivation of the ELBO with Structured Latent Variables

### Appendix A.1. Evidence for Lower Bound CVAE with Structured Latent Variables

To address the limitations of simplistic priors and insufficient dependency modeling between motion and spatial context, we enhance the standard CVAE by incorporating structured latent variables, as introduced in Equation (1).

Given the inputs X and $M_{0}$ and the latent variables $z_{V}$ and $z_{C}$, we assume that the vehicle motion modality output Y and the map information modality output $M_{F}$ are conditionally independent. Under this assumption, the reconstruction error in the first term on the right-hand side of Equation (1) can be decomposed into two separate parts. Based on the above assumption, the reconstruction error term can be rewritten as follows:

(A1) $$\begin{array}{cc} \; & E_{q(z_{V},z_{C}|X,M_{0},Y,M_{F})}\left[\log p(Y,M_{F}|z_{V},z_{C},X,M_{0})\right]\\ = & E_{q(z_{V},z_{C}|X,M_{0},Y,M_{F})}\left[\log p(Y|z_{V},z_{C},X,M_{0})\right]\\ \; & +E_{q(z_{V},z_{C}|X,M_{0},Y,M_{F})}\left[\log p(M_{F}|z_{V},z_{C},X,M_{0})\right]. \end{array}$$

Equation (A1) decomposes the original joint probability density function into two independent conditional probability density functions, demonstrating that the decomposition of the vehicle trajectory prediction task and the lane sequence prediction task is feasible. This supports the use of two independent decoders or functions to separately predict Y and $M_{F}$.

Considering the decomposition of latent variables $z_{V}$ and $z_{C}$ introduced in Section 3.2, we let $z_{V}=\left(z_{\mathrm{VP}},z_{S}\right)$ and $z_{C}=\left(z_{\mathrm{CP}},z_{S}\right),$ where $z_{\mathrm{VP}}$ and $z_{\mathrm{CP}}$ encode the factors specific to vehicle dynamics and lane-level map semantics and $z_{S}$ represents the latent features shared between the two modalities. Based on this decomposition, we now revisit the reconstruction term in Equation (A1), which expresses the expected log-likelihood of motion prediction conditioned on the latent variables. Substituting the structured form of the latent space yields

(A2) $$\begin{array}{cc} \; & E_{q(z_{V},z_{C}|X,M_{0},Y,M_{F})}\left[\log p(Y|z_{V},z_{C},X,M_{0})\right]\\ = & E_{q(z_{\mathrm{VP}},z_{\mathrm{CP}},z_{S}|X,M_{0},Y,M_{F})}\left[\log p(Y|z_{\mathrm{VP}},z_{\mathrm{CP}},z_{S},X,M_{0})\right]. \end{array}$$

Given the conditional independence assumption that

(A3) $$p\left(Y|z_{\mathrm{VP}},z_{\mathrm{CP}},z_{S},X,M_{0}\right)=p\left(Y|z_{\mathrm{VP}},z_{S},X,M_{0}\right),$$

we can simplify the above expression to

(A4) $$E_{q(z_{\mathrm{VP}},z_{S}|X,M_{0},Y,M_{F})}\left[\log p(Y|z_{\mathrm{VP}},z_{S},X,M_{0})\right].$$

Similarly, since the scene map output $M_{F}$ and the vehicle-specific latent variable $z_{\mathrm{VP}}$ are conditionally independent given $z_{\mathrm{CP}},z_{S},X,$ and $M_{0}$, the second term in the decomposition of Equation (A1) can be simplified as follows:

(A5) $$\begin{array}{cc} \; & E_{q(z_{V},z_{C}|X,M_{0},Y,M_{F})}\left[\log p(M_{F}|z_{V},z_{C},X,M_{0})\right]\\ = & E_{q(z_{\mathrm{CP}},z_{S}|X,M_{0},Y,M_{F})}\left[\log p(M_{F}|z_{\mathrm{CP}},z_{S},X,M_{0})\right]. \end{array}$$

To complete the derivation, we next consider the KL term in the ELBO, where the independence assumption allows for a tractable decomposition. Assuming independence between $z_{\mathrm{VP}}$ and $z_{\mathrm{CP}}$, the KL term in Equation (1) can be decomposed as follows:

(A6) $$\begin{array}{cc} \; & \mathrm{KL}\left[q\left(z_{V},z_{C}|X,M_{0},Y,M_{F}\right)\|\;p\left(z_{V},z_{C}|X,M_{0}\right)\right]\\ = & \mathrm{KL}\left[q\left(z_{\mathrm{VP}},z_{S}|X,M_{0},Y,M_{F}\right)\|\;p\left(z_{\mathrm{VP}},z_{S}|X,M_{0}\right)\right]\\ \; & +\mathrm{KL}\left[q\left(z_{\mathrm{CP}},z_{S}|X,M_{0},Y,M_{F}\right)\|\;p\left(z_{\mathrm{CP}},z_{S}|X,M_{0}\right)\right]. \end{array}$$

Combining the results from Equations (A1) to (A6), the lower bound for the multi-source CVAE with structured latent variables is given by Equation (2) in Section 3.3.

### Appendix A.2. Incorporating Total Correlation Regularization

Despite the inductive bias introduced by the structured latent variable model to separate individual-specific ($z_{\mathrm{VP}}$, $z_{\mathrm{CP}}$) and shared ($z_{S}$) representations, residual statistical entanglement may still persist among these latent components. Such dependencies can result in redundant encoding and compromise the disentanglement of the latent space.

To further encourage independent factorization, we incorporate a TC decomposition term into the variational objective. The TC regularizer penalizes mutual dependence among the latent dimensions, promoting a more disentangled and interpretable latent structure.

The KL term related to $z_{V}$ in Equation (2) can be expressed in its TC-decomposed form:

(A7) $$\begin{array}{cc} \; & \mathrm{KL}\left[q\left(z_{\mathrm{VP}},z_{S}|X,M_{0},Y,M_{F}\right)\|\;p\left(z_{\mathrm{VP}},z_{S}|X,M_{0}\right)\right]\\ = & \mathrm{KL}\left[q\left(z_{\mathrm{VP}},z_{S}|X,M_{0},Y,M_{F}\right)\|\;q\left(z_{\mathrm{VP}}|X,Y\right)q\left(z_{S}|X,M_{0},Y,M_{F}\right)\right]\\ \; & +\mathrm{KL}\left[q\left(z_{\mathrm{VP}}|X,Y\right)\|\;p\left(z_{\mathrm{VP}}|X\right)\right]+\mathrm{KL}\left[q\left(z_{S}|X,M_{0},Y,M_{F}\right)\|\;p\left(z_{S}|X,M_{0}\right)\right]. \end{array}$$

Similarly, the KL term related to $z_{C}$ in Equation (2) can be decomposed as follows:

(A8) $$\begin{array}{cc} \; & \mathrm{KL}\left[q\left(z_{\mathrm{CP}},z_{S}|X,M_{0},Y,M_{F}\right)\|\;p\left(z_{\mathrm{CP}},z_{S}|X,M_{0}\right)\right]\\ = & \mathrm{KL}\left[q\left(z_{\mathrm{CP}},z_{S}|X,M_{0},Y,M_{F}\right)\|\;q\left(z_{\mathrm{CP}}|M_{0},M_{F}\right)q\left(z_{S}|X,M_{0},Y,M_{F}\right)\right]\\ \; & +\mathrm{KL}\left[q\left(z_{\mathrm{CP}}|M_{0},M_{F}\right)\|p\left(z_{\mathrm{CP}}|M_{0}\right)]+\mathrm{KL}[q\left(z_{S}|X,M_{0},Y,M_{F}\right)\|\;p\left(z_{S}|X,M_{0}\right)\right]. \end{array}$$

Considering the independence between $z_{\mathrm{VP}}$ and the map information $M_{0}$, the terms in Equations (A7) and (A8) that involve only the latent variable $z_{\mathrm{VP}}$ are not conditional on $M_{0}$ or $M_{F}$. Similarly, given the independence between $z_{\mathrm{CP}}$ and vehicle motion, the terms involving only $z_{\mathrm{CP}}$ do not depend on X or Y. Incorporating the decompositions from Equations (A7) and (A8) into Equation (2) yields the ELBO with TC regularization shown in Equation (3).

## References

[1] Zhao T., Xu Y., Monfort M., Choi W., Baker C., Zhao Y., Wang Y., Wu Y.N. Multi-agent tensor fusion for contextual trajectory prediction. Proceedings of the IEEE/CVF Conference on Computer Vision and Pattern Recognition (CVPR).

[2] Yuan Y., Weng X., Ou Y., Kitani K. AgentFormer: Agent-aware transformers for socio-temporal multi-agent forecasting. Proceedings of the IEEE/CVF International Conference on Computer Vision (ICCV).

[3] Liang M., Yang B., Hu R., Chen Y., Liao R., Feng S., Urtasun R. Learning lane graph representations for motion forecasting. Proceedings of the European Conference on Computer Vision (ECCV).

[4] Ngiam J., Caine B., Vasudevan V., Zhang Z., Chiang H.L., Ling J., Roelofs R., Bewley A., Liu C., Venugopal A. (2021). Scene transformer: A unified architecture for predicting multiple agent trajectories. arXiv.

[5] Goodfellow I.J., Pouget-Abadie J., Mirza M., Xu B., Warde-Farley D., Ozair S., Courville A., Bengio Y. (2014). Generative adversarial networks. arXiv.

[6] Kingma D.P. (2013). Auto-encoding variational bayes. arXiv.

[7] Sohn K., Lee H., Yan X. (2015). Learning structured output representation using deep conditional generative models. Adv. Neural Inf. Process. Syst..

[8] Huang R., Xue H., Pagnucco M., Salim F., Song Y. (2023). Multimodal trajectory prediction: A survey. arXiv.

[9] Kim H., Mnih A. Disentangling by factorising. Proceedings of the International Conference on Machine Learning.

[10] Deo N., Trivedi M.M. Convolutional social pooling for vehicle trajectory prediction. Proceedings of the IEEE Conference on Computer Vision and Pattern Recognition Workshops (CVPRW), Salt Lake City.

[11] Li X., Ying X., Chuah M.C. (2020). Grip++: Enhanced graph-Based interaction-aware trajectory prediction for autonomous driving. arXiv.

[12] Gao J., Sun C., Zhao H., Shen Y., Anguelov D., Li C., Schmid C. Vectornet: Encoding HD maps and agent dynamics from vectorized representation. Proceedings of the IEEE Conference on Computer Vision and Pattern Recognition (CVPR).

[13] Zhou Z., Ye L., Wang J., Wu K., Lu K. Hivt: Hierarchical vector transformer for multi-agent motion prediction. Proceedings of the IEEE Conference on Computer Vision and Pattern Recognition (CVPR).

[14] Deo N., Wolff E., Beijbom O., Faust A., Hsu D., Neumann G. (2021). Multimodal trajectory prediction conditioned on lane-Graph traversals. Proceedings of the 5th Conference on Robot Learning (CoRL).

[15] Gupta A., Johnson J., Li F., Savarese S., Alahi A. Social gan: Socially acceptable trajectories with generative adversarial networks. Proceedings of the IEEE Conference on Computer Vision and Pattern Recognition (CVPR).

[16] Salzmann T., Ivanovic B., Chakravarty P., Pavone M. (2021). Trajectron++: Dynamically-feasible trajectory forecasting with heterogeneous data. arXiv.

[17] Varadarajan B., Hefny A., Srivastava A., Refaat K.S., Nayakanti N., Cornman A., Chen K., Douillard B., Lam C.P., Anguelov D. Multipath++: Efficient information fusion and trajectory aggregation for behavior prediction. Proceedings of the IEEE International Conference on Robotics and Automation (ICRA).

[18] Zhao H., Gao J., Lan T., Sun C., Sapp B., Varadarajan B., Shen Y., Shen Y., Chai Y., Schmid C., Kober J., Ramos F., Tomlin C. (2021). TNT: Target-driven trajectory prediction. Proceedings of the Conference on Robot Learning (CoRL).

[19] Chiara L.F., Coscia P., Das S., Calderara S., Cucchiara R., Ballan L. Goal-driven self-attentive recurrent networks for trajectory prediction. Proceedings of the IEEE/CVF Conference on Computer Vision and Pattern Recognition Workshops (CVPRW).

[20] Zhang Y., Su J., Guo H., Li C., Lv P., Xu M. (2024). S-CVAE: Stacked CVAE for trajectory prediction with incremental greedy region. IEEE Trans. Intell. Transp. Syst..

[21] Chen R.T.Q., Li X., Grosse R., Duvenaud D., Bengio S., Wallach H., Larochelle H., Grauman K., Cesa-Bianchi N., Garnett R. (2018). Isolating Sources of Disentanglement in Variational Autoencoders. Advances in Neural Information Processing Systems 31, Proceedings of the International Conference on Neural Information Processing Systems 2018, Montreal, QC, Canada, 3–8 December 2018.

[22] Guzmán-rivera A., Batra D., Kohli P., Pereira F., Burges C., Bottou L., Weinberger K. (2012). Multiple choice learning: Learning to produce multiple structured outputs. Advances in Neural Information Processing Systems 25, Proceedings of the Advances in Neural Information Processing Systems 2012, Lake Tahoe, NV, USA, 3–6 December 2012.

[23] Caesar H., Bankiti V., Lang A.H., Vora S., Liong V.E., Xu Q., Krishnan A., Pan Y., Baldan G., Beijbom O. NuScenes: A multimodal dataset for autonomous driving. Proceedings of the IEEE Conference on Computer Vision and Pattern Recognition (CVPR).

[24] Phan-Minh T., Grigore E.C., Boulton F.A., Beijbom O., Wolff E.M. Covernet: Multimodal behavior prediction using trajectory sets. Proceedings of the IEEE Conference on Computer Vision and Pattern Recognition (CVPR).

[25] Cui H., Radosavljevic V., Chou F., Lin T., Nguyen T., Huang T., Schneider J., Djuric N. Multimodal trajectory predictions for autonomous driving using deep convolutional networks. Proceedings of the International Conference on Robotics and Automation (ICRA).

[26] Chai Y., Sapp B., Bansal M., Anguelov D. Multipath: Multiple probabilistic anchor trajectory hypotheses for behavior prediction. Proceedings of the Conference on Robot Learning.

